# Third dose vaccination with mRNA-1273 or BNT162b2 vaccines improves protection against SARS-CoV-2 infection

**DOI:** 10.1093/pnasnexus/pgac042

**Published:** 2022-04-28

**Authors:** Michiel J M Niesen, Robert Matson, Arjun Puranik, John C O'Horo, Colin Pawlowski, Celine Vachon, Douglas Challener, Abinash Virk, Melanie Swift, Leigh Speicher, Joel Gordon, Holly Geyer, Patrick J Lenehan, A J Venkatakrishnan, Venky Soundararajan, Andrew Badley

**Affiliations:** nference, Cambridge, MA 02139, USA; nference, Cambridge, MA 02139, USA; nference, Cambridge, MA 02139, USA; Division of Infectious Diseases, Mayo Clinic, Rochester, MN 55905, USA; Division of Pulmonary and Critical Care Medicine, Mayo Clinic, Rochester, MN 55905, USA; nference, Cambridge, MA 02139, USA; Division of Quantitative Health Science, Mayo Clinic, Rochester, MN 55905, USA; Division of Infectious Diseases, Mayo Clinic, Rochester, MN 55905, USA; Division of Infectious Diseases, Mayo Clinic, Rochester, MN 55905, USA; Division of Preventive, Occupational and Aerospace Medicine, Mayo Clinic, Rochester, MN 55902, USA; Division of General Internal Medicine, Mayo Clinic, Jacksonville, FL 32224, USA; Department of Family Medicine, Mayo Clinic Health System, Mankato, MN 56001, USA; Division of Hospital Internal Medicine, Mayo Clinic, Phoenix, AZ 85054, USA; nference, Cambridge, MA 02139, USA; nference, Cambridge, MA 02139, USA; nference, Cambridge, MA 02139, USA; Division of Infectious Diseases, Mayo Clinic, Rochester, MN 55905, USA; Department of Molecular Medicine, Mayo Clinic, Rochester, MN 55905, USA

**Keywords:** COVID-19, Vaccine Effectiveness, Electronic Health Records, Booster, EHR

## Abstract

As of 2021 November 29, booster vaccination against SARS-CoV-2 infection has been recommended for all individuals aged 18 years and older in the United States. A key reason for this recommendation is the expectation that a booster vaccine dose can alleviate observed waning of vaccine effectiveness (VE). Although initial reports of booster effectiveness have been positive, the level of protection from booster vaccination is unclear. We conducted two studies to assess the impact of booster vaccination, with BNT162b2 or mRNA-1273, on the incidence of SARS-CoV-2 infection between August and December 2021. We first compared SARS-CoV-2 infection incidence in cohorts of 3-dose vaccine recipients to incidence in matched cohorts of 2-dose vaccine recipients (cohort size = 24,539 for BNT162b2 and 14,004 for mRNA-1273). Additionally, we applied a test-negative study design to compare the level of protection against symptomatic infection in 3-dose recipients to that observed in recent 2-dose primary vaccine series recipients. The 3-dose recipients experienced a significantly lower incidence rate of SARS-CoV-2 infection than the matched 2-dose cohorts (BNT162b2 Incidence Rate Ratio: 0.11, 95% CI: 0.09 to 0.13 and mRNA-1273 IRR: 0.11, 95% CI: 0.08 to 0.15). Results from the test-negative study showed the third vaccine dose mitigated waning of VE, with the risk of symptomatic infection in 3-dose recipients being comparable to that observed 7 to 73 days after the primary vaccine series. These results show that 3-dose vaccine regimens with BNT162b2 or mRNA-1273 are effective at reducing SARS-CoV-2 infection and support the widespread administration of booster vaccine doses.

Significance Statement Booster vaccination with approved COVID-19 vaccines has been recommended for all individuals aged 18 years and older in the United States. In this study, we retrospectively analyzed electronic health records to assess the benefit of a third vaccine dose in preventing SARS-CoV-2 infection and COVID-19 associated hospitalization, compared to the standard 2-dose regimen. A third vaccine dose was beneficial for all age groups and patient subpopulations tested, including immunocompromised individuals, individuals aged 18 to 49 years, and individuals aged 50 years and over.

## Introduction

BNT1612b2 and mRNA-1273 are messenger RNA (mRNA) vaccines encoding a prefusion stabilized form of the SARS-CoV-2 Spike glycoprotein (1, 2). These were designed and tested for use in a 2-dose serial vaccination strategy, with a recommended separation between doses of 21 and 28 days for BNT162b2 and mRNA-1273, respectively. Clinical trials and real-world studies showed that this dosing strategy provided robust protection against symptomatic and severe COVID-19 (3–6). However, for some immunocompromised individuals, an additional (third) dose was recommended to achieve the full level of protection (7). Further, the declining effectiveness of these vaccines in preventing symptomatic illness over time (8–14) has prompted recommendations for a booster vaccine dose in the general population (15). Booster vaccination with the Food and Drug Administration (FDA) authorized mRNA-based COVID-19 vaccines involves a third vaccine dose, recommended to be administered at least 5 months after the initial 2-dose regimen. This booster dose is intended to raise protection against infection, severe disease, and death, and is especially recommended for populations that are at high risk for severe disease, such as immunocompromised individuals and individuals with solid tumors (16, 17).

Preliminary studies have shown that a booster dose can increase serum antibody titers and protection against infection (18–23). Other studies have demonstrated the safety of booster vaccination, showing a similar adverse event profile as the first two doses (24–28). As more booster doses are administered, including in the general population and using multiple vaccine brands, it is important to monitor the potential benefits and risks. Here, we quantitatively assess whether booster vaccination with the FDA-authorized mRNA-based COVID-19 vaccines effectively reduces the risk of SARS-CoV-2 infection and COVID-19 associated hospitalization.

## Results

### BNT162b2 and mRNA-1273 3-dose vaccine regimens are associated with lower rates of SARS-CoV-2 infection compared to 2-dose vaccine regimens

**Fig. 1.**
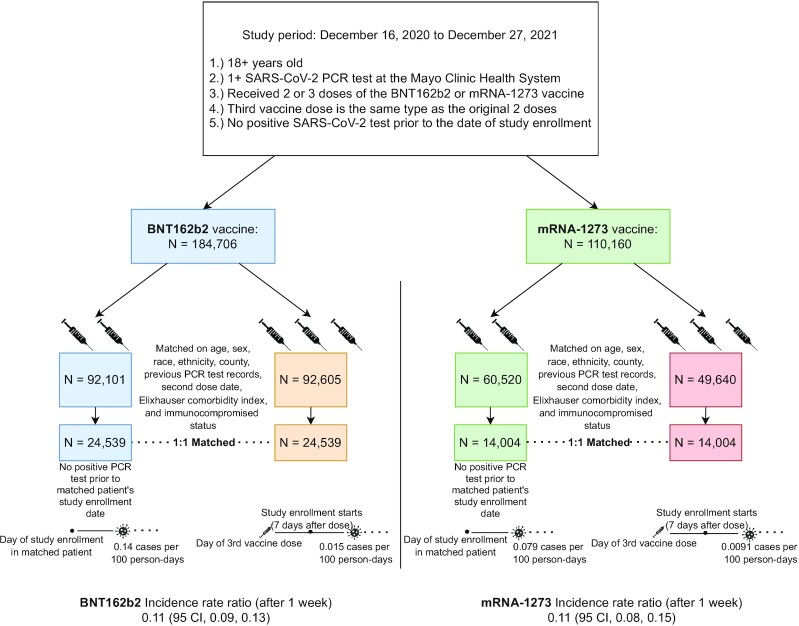
Schematic depiction of the cohort study design. We performed a matched cohort study to assess the effectiveness of a 3-dose vaccine regimen compared to the initial 2-dose regimen. The 3-dose recipients that meet all study inclusion criteria were 1:1 matched with 2-dose recipients on the date of their second dose, demographic characteristics, and immunocompromised status. Cohorts were established separately for the two vaccines.

**Fig. 2.**
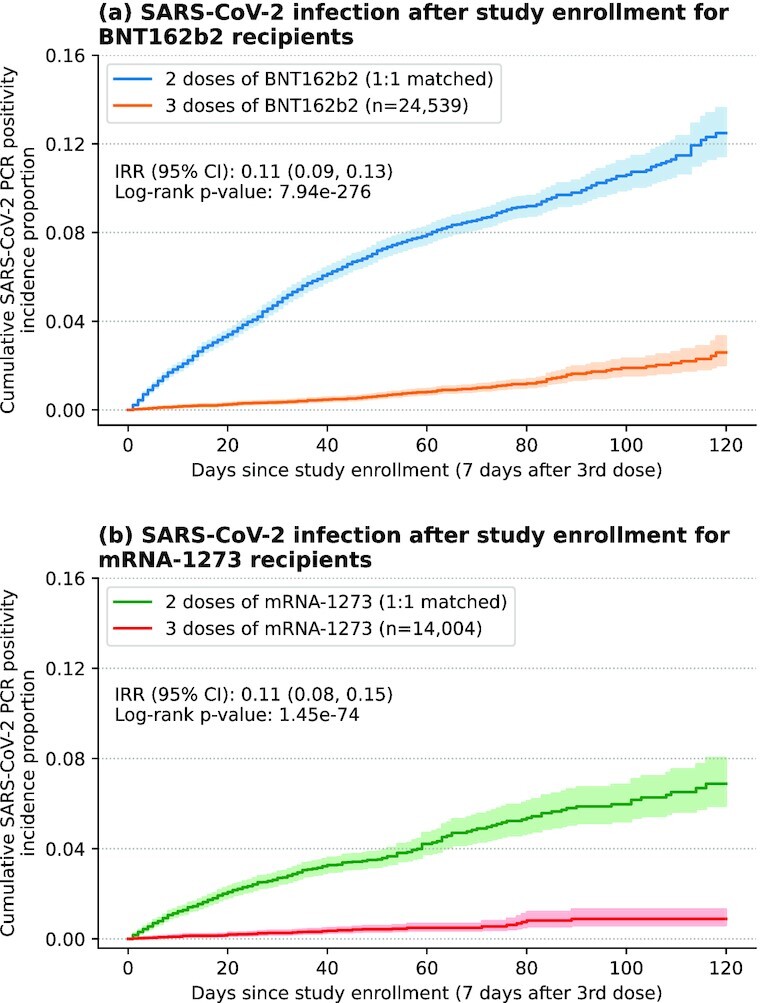
Kaplan–Meier analysis of the relative risk of SARS-CoV-2 infection. Shown is the cumulative incidence of positive SARS-CoV-2 PCR tests; (a) for 3-dose BNT162b2 recipients (orange) and their 1:1 matched 2-dose recipients (blue), and (b) for 3-dose mRNA-1273 recipients (red) and their 1:1 matched 2-dose recipients (green). Shaded regions correspond to 95% CI.

To evaluate the benefit of booster vaccination for BNT162b2 and mRNA-1273, we compared the incidence rates of SARS-CoV-2 infection after study enrollment in a 3-dose cohort and a matched 2-dose cohort for each vaccine type (Fig. 1). The study enrollment date corresponds to the date of the third vaccine dose for the 3-dose cohort. For each vaccine type, the 3-dose and 2-dose cohorts were well-matched across a range of clinical covariates, including demographics (age, sex, race, and ethnicity), geography (county of primary residence), immunocompromised status, number of previous PCR tests, and the calendar week of second dose (Table S1, Supplementary Material). For each vaccine, Kaplan–Meier analysis showed that the cumulative SARS-CoV-2 incidence rate after study enrollment was significantly lower in each 3-dose cohort compared to the matched 2-dose cohort (BNT162b2 log-rank *P*-value: < 0.001;  mRNA-1273 log-rank *P*-value: < 0.001; Fig. 2). Similar results were found for the incidence of symptomatic infections (Figure S1, Supplementary Material).

Furthermore, for each vaccine type the 3-dose cohort had significantly lower rates of SARS-CoV-2 infection for all time periods considered following study enrollment, including days 1 to 14, days 15 to 28, days 29 to 42, days 43 to 56, day 1 onwards, day 8 onwards, day 15 onwards, and day 22 onwards. For the time period day 15 onwards, the incidence rate of SARS-CoV-2 infection was 0.0154 cases per 100 person days in the 3-dose BNT162b2 cohort vs. 0.117 cases per 100 person days in the matched 2-dose BNT162b2 cohort (IRR: 0.13, 95% CI: [0.11,0.16]; Table S2, Supplementary Material). Similarly, for the same time period, the incidence rate was 0.0087 cases per 100 person days in the 3-dose mRNA-1273 cohort vs. 0.063 cases per 100 person days in the matched 2-dose mRNA-1273 cohort (IRR: 0.14, 95% CI: [0.09,0.20]; Table S3, Supplementary Material).

### BNT162b2 and mRNA-1273 3-dose vaccine regimens are effective in immunosuppressed individuals and in distinct age groups

We repeated the above analysis for subpopulations of immunosuppressed individuals and individuals at least 50 years old to evaluate the impact of the third dose. Among immunocompromised individuals, the cumulative SARS-CoV-2 incidence rate was significantly lower in the 3-dose BNT162b2 cohort compared to the matched 2-dose BNT162b2 cohort (IRR: 0.16, 95% CI: [0.02, 0.70], log-rank *P*-value: 0.003; Figure S2a, Supplementary Material). For the mRNA-1273 vaccine, the cumulative SARS-CoV-2 incidence rates was lower in the 3-dose immunocompromised cohort compared to the matched 2-dose immunocompromised cohorts, however, the difference was not statistically significant (IRR: 0.47, 95% CI: [0.04, 3.30], log-rank *P*-value: 0.371; Figure S2b, Supplementary Material). We further assessed the effectiveness of a third dose in individuals stratified by age. For both BNT162b2 and mRNA-1273, the 3-dose cohorts showed significantly lower incidence rates of SARS-CoV-2 infection compared with the 2-dose cohorts among individuals at least 50 years old (Figure S3, Supplementary Material) and individuals between ages 18 and 49 (Figure S4, Supplementary Material).

### BNT162b2 and mRNA-1273 3-dose vaccine regimens are effective in protecting against severe COVID-19

**Fig. 3.**
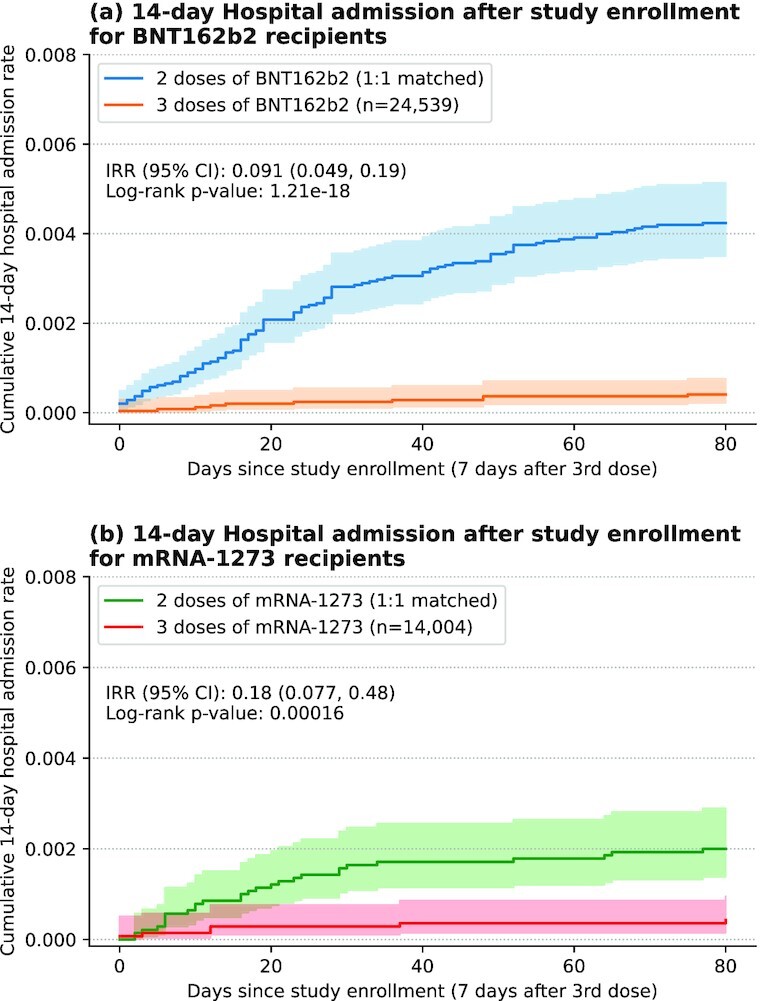
Kaplan–Meier analysis of the relative risk of SARS-CoV-2 infection and subsequent hospitalization. SARS-CoV-2 infection and subsequent hospitalization is defined as a positive PCR test after study enrollment along with hospitalization within 14 days. Shown are the cumulative rates for: (a) 3-dose BNT162b2 recipients (orange) and their 1:1 matched 2-dose controls (blue), and (b) 3-dose mRNA-1273 recipients (red) and their 1:1 matched 2-dose controls (green). Shaded regions correspond to 95% CI.

To assess protection against severe COVID-19, we also considered the outcomes of 14-day COVID-19 associated hospitalization and emergency department (ED) admission. For both vaccines, the rates of SARS-CoV-2 infection and subsequent hospitalization were significantly lower in the 3-dose cohort compared to the 2-dose cohort (BNT162b2 IRR: 0.09, 95% CI: [0.05, 0.19], log-rank *P*-value: < 0.001;  mRNA-1273 IRR: 0.18, 95% CI: [0.08, 0.48], log-rank *P*-value: < 0.001; Fig. 3).  We observed similar trends for the rates of SARS-CoV-2 infection and subsequent ED admission (BNT162b2 IRR: 0.07, 95% CI: [0.04, 0.13], log-rank *P*-value: < 0.001;  mRNA-1273 IRR: 0.14, 95% CI: [0.06, 0.36], log-rank *P*-value: < 0.001; Figure S5, Supplementary Material).

### BNT162b2 and mRNA-1273 3-dose vaccine regimens are associated with lower rates of symptomatic SARS-CoV-2 infection 4 to 11 months following full vaccination

**Fig. 4.**
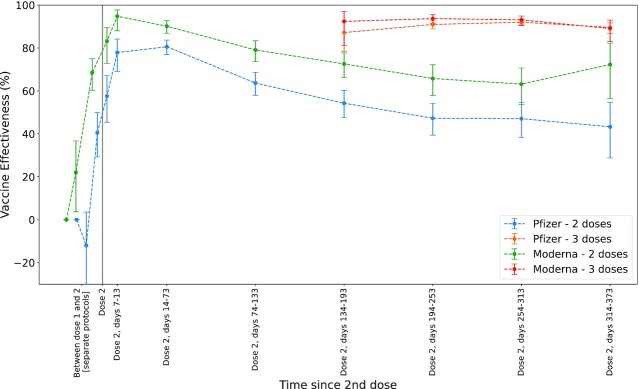
Estimated VE over time. VE was estimated based on the odds ratio of positive SARS-CoV-2 PCR tests and negative SARS-CoV-2 PCR tests, relative to this odds ratio for individuals that just received a first vaccine dose (1 to 6 days after dose 1, left most datapoint). VE was estimated for: 2-dose BNT162b2 recipients (blue), 3-dose BNT162b2 recipients (orange), 2-dose mRNA-1273 recipients (green), and 3-dose mRNA-1273 recipients (red). Error bars indicate 95% CI.

To assess the duration of protection for 3-dose BNT162b2 and mRNA-1273 vaccine regimens, we used a test-negative study design to evaluate vaccine effectiveness (VE) for the 2-dose and 3-dose cohorts for a range of time intervals relative to the first and second doses (Methods). For each vaccine type, we used the odds of symptomatic SARS-CoV-2 infection in days 1 to 6 following the first dose as the baseline to estimate VE for a variety of time intervals. For the 2-dose BNT162b2 cohort, we observed that VE peaks at 80.6% (95% CI: [76.9%, 83.7%]) during the 60-day time period following the second dose (14 to 73 days following full vaccination) and declined to 43.3% (95% CI: [28.8%, 54.7%]) during the final time period (314 to 355 days after the second dose; Fig. 4; Table S4, Supplementary Material). A third dose of BNT162b2 was highly effective irrespective of time since the second dose, with an estimated VE of 89.6% (95% CI: [86.9%, 91.7%]) during the final time period (see Fig. 4; Table S4, Supplementary Material).

We observed a similar trend for mRNA-1273. For the 2-dose mRNA-1273 regimen, VE peaked at 94.8% (95% CI: [88.0%, 97.8%]) during the time period 7 to 13 days following the second dose and declined to 63.2% (95% CI: [53.7%, 70.7%]) during the penultimate time period (days 254 to 313 after second dose; Fig. 4; Table S5, Supplementary Material). A third dose of mRNA-1273 was highly effective irrespective of time since the second dose, with an estimated VE of 89.1% (95% CI: [82.9%, 93.0%]) for the final time period (314 to 339 days after second dose; Fig. 4; Table S5, Supplementary Material).

## Discussion

In this study, we showed that both BNT162b2 and mRNA-1273 3-dose vaccine regimens are associated with reduced incidence of SARS-CoV-2 infection, predominantly during a time in which the Delta variant was dominant. For each vaccine type, the incidence rate of SARS-CoV-2 2 weeks after the third dose was significantly lower than the incidence rate of SARS-CoV-2 for the 2-dose cohort, including in immunocompromised hosts. This approximately 10-fold decrease in incidence provides strong evidence that the booster vaccine doses offer strong protection against infection with SARS-CoV-2 (especially the Delta variant) and is in line with earlier estimates of BNT162b2 booster effectiveness from Israel (29). Furthermore, the estimates of VE for the 3-dose regimens in the time period 4 to 11 months following full vaccination are comparable to the estimates of VE for the 2-dose regimens in the time period 1 to 2 months following full vaccination and those reported in initial clinical trials (5, 6). Mechanistically, it has been shown that third doses of BNT162b2 and mRNA-1273 elicit increased serum neutralizing antibody titers (23, 25, 27, 28, 30, 31). Prolonged transudation of these elevated titers into nasopharyngeal secretions of the upper airways may mediate the protective effect of booster vaccination, which could explain the strong effect sizes observed in this study. Given the recent surge in new SARS-CoV-2 infections and large disease burden of the COVID-19 pandemic in the United States and the rest of the world (32), this study underscores the need for large-scale vaccination efforts including full vaccination and booster doses for the mRNA COVID-19 vaccines. The VE against Omicron variant is yet to be fully elucidated, however, initial evidence suggests that both BNT162b2 and mRNA-1273 are less effective against infection with Omicron in comparison to Delta (33–35). This study did not have enough Omicron cases to draw a meaningful conclusion.

Although we assess the real-world effectiveness of both BNT162b2 and mRNA-1273 vaccines in this study, we cannot directly compare the estimates of VE because the underlying vaccinated populations have different clinical characteristics. For example, 13.7% of the matched BNT162b2 cohorts have primary residences in Florida, while 20.4% of the matched mRNA-1273 cohorts have primary residences in Florida (Table S1, Supplementary Material). Since SARS-CoV-2 exposure is variable across states, the VE estimates for each vaccine type will be different so we cannot conclude if one mRNA 3-dose regimen is more protective than the other. However, it should be noted that both 3-dose regimens are highly effective within their cohorts, with effectiveness point estimates between 87.2% and 92.0% for BNT162b2 and 92.4% and 93.7% for mRNA-1273 (Fig. 4; Tables S4 to S5, Supplementary Material). Another caveat is that the VE estimates in this study were not computed using an unvaccinated control cohort. This is because at the time of the study (late December 2021), there were few unvaccinated individuals in the Mayo Clinic EHR dataset, and the size of this unvaccinated cohort was too small to perform a propensity matched analysis in comparison with the 3-dose and 2-dose cohorts. To estimate the effectiveness for each vaccine regimen in this study, we took the baseline SARS-CoV-2 incidence rate for each cohort to be the SARS-CoV-2 incidence rate for that cohort during the time period 1 to 6 days following the first vaccine dose. For example, for the 3-dose mRNA-1273 cohort, we estimated the VE using the baseline rate of SARS-CoV-2 incidence during the time period 1 to 6 days following the first dose of mRNA-1273. Other real-world evidence studies of COVID-19 VE may use alternate definitions for the baseline SARS-CoV-2 incidence rates, which could lead to slightly different estimates of VE.

There are several limitations of this study. First, the study population was predominantly white and non-Hispanic. Follow-up studies are warranted in health systems with a more demographically diverse patient population. Second, the timing between the second and third doses was not standardized and is highly variable across the study population. As a result, although this study shows that booster vaccination is strongly effective in reducing SARS-CoV-2 incidence, it is not clear if and how the timing of the booster dose impacts VE. Third, it is possible that vaccination status was incorrectly captured for some of the individuals in the study population. For example, some individuals in the 2-dose cohort may have received a booster dose, which is not recorded in the database. However, we expect that this source of error will be relatively small because state-level COVID-19 vaccine registry data is used to confirm vaccination status for individuals in this study. For mRNA-1273 recipients, it is also expected that recent recipients received a half-dose booster, based on updated guidelines (36), while earlier recipients of mRNA-1273 likely received a full dose owing to the dose being an “additional dose” instead of the half-dose booster (latter approved for use in October 2021). Fourth, we did not consider cohorts with mixed vaccine regimens (e.g. BNT162b2 2-dose + mRNA-1273 booster or mRNA-1273 2-dose + BNT162b2 booster) due to limited sample sizes. Follow-up studies are needed to assess the real-world effectiveness of mixed vaccine regimens, including combinations of BNT162b2, mRNA-1273, and Ad.26.COV2.S, and to assess booster protection against severe disease. Follow-up studies should also evaluate the impact of new variants, such as the Omicron variant, which had only just emerged during this study. Recently, boosters have been recommended for ages 12 and older. Recommendations in effect during the timeframe of our observational study therefore limits our findings to adults age 18 and older. Additional studies in younger age groups will be needed. Finally, booster vaccination recommendations must consider both benefits and potential harms, and our study did not evaluate adverse events. Our findings add to the growing body of literature supporting the effectiveness of booster vaccination, while other studies have not found unexpected patterns of adverse reactions.

Overall, this study provides support for the administration of booster vaccine doses per the current guidelines for adults. The data show that a booster dose can mitigate waning VE, restoring levels of protection that are comparable to immediately following the 2-dose primary series.

## Methods

### Study population and setting

We implemented both a cohort study and test-negative study design to retrospectively assess the effectiveness of BNT162b2 and mRNA-1273 booster vaccination. Cohorts were sampled from two underlying study populations including individuals who received their third dose of the BNT162b2 or mRNA-1273 vaccine between 2021 August 12 and 2021 December 27, and individuals who only received two doses of the BNT162b2 or mRNA-1273 vaccine between 2020 December 16 and 2021 December 27, and who subsequently underwent SARS-CoV-2 polymerase chain reaction (PCR) testing at the Mayo Clinic. This study was reviewed by the Mayo Clinic Institutional Review Board (IRB) and deemed exempt from the requirement for IRB approval (IRB#: 20–003278). Study participants were excluded if they did not have a research authorization on file. Inclusion and exclusion criteria for both studies were defined as follows:

Inclusion criteria for both studies:

1. Age greater than or equal to 18 years as of 2020 December 16.
2. Received two or three doses of the BNT162b2 or mRNA-1273 vaccine per-protocol, with the first dose administered on or after 2020 December 16, and the third dose administered on or after 2021 August 12 (see Figure S6 (Supplementary Material) for vaccine dose timing). For the first two doses, per-protocol BNT162b2 vaccination was defined as the first and second doses administered 18 to 28 days apart, and per-protocol mRNA-1273 vaccination was defined as the first and second doses administered 25 to 35 days apart. For the third dose, per-protocol vaccination (for either BNT162b2 or mRNA-1273) was defined as a homologous third dose administered at least 120 days after the second dose. Receiving any other COVID-19 vaccine (i.e. a heterologous primary or booster vaccination series) was considered off-protocol.

Note that for the test-negative study, we applied the on-protocol inclusion criteria *at the time of the test*—if the individual went off-protocol after the test, that test is still included.

Cohort study exclusion criteria:

1. Any positive SARS-CoV-2 PCR test prior to the date of study enrollment (defined as 7 days after the third dose).
2. Did not receive an additional booster (i.e. an additional COVID-19 vaccine dose after the third dose).

Test-negative study exclusion criteria:

1. Any prior positive SARS-CoV-2 PCR test.
2. Third dose within 13 days before the test, or within 3 days after the test.

Study participants were divided into four cohorts based on the vaccine series administered to them. After the implementation of the inclusion and exclusion criteria, the BNT162b2 cohort study population included 92,605 eligible 3-dose individuals and 92,101 eligible 2-dose individuals and the mRNA-1273 cohort study population included 49,640 eligible 3-dose individuals and 60,520 eligible 2-dose individuals.

### Cohort study design

We constructed a 2-dose cohort matched to the 3-dose cohort with respect to potential confounding factors. The following potential confounders were included as covariates for matching: demographic characteristics (age, sex, race, and ethnicity), geography (county of primary residence), number of negative SARS-CoV-2 PCR tests between 2020 December 16 and 2021 December 27, number of comorbidities derived from the Elixhauser Comorbidity Index (37), immunosuppressant usage, and date of the second vaccine dose. To determine Elixhauser comorbidities, we considered ICD codes in the 5 years leading up to the second dose date. To determine immunosuppressant usage, we considered orders for immunosuppressant medications drug class WHO ATC LO4A (38) during the 1 year leading up to the second dose date. Several of the covariates were bucketed prior to the matching procedure. Age was bucketed into the following categories prior to matching: 18 to 24, 25 to 34, 35 to 44, 45 to 54, 55 to 64, 65 to 74, 75 to 84, and 85+ years old. The number of Elixhauser comorbidities was bucketed into the following categories prior to matching: 0, 1 to 4, 5 to 9, and 10+. Date of the second vaccine dose was matched with a buffer of +/− 7 days. All other covariates were matched exactly.

Using these covariates, we matched each of the 92,605 individuals in the previously defined 3-dose BNT162b2 cohort study population with one eligible 2-dose BNT162b2 individual and each of the 49,640 individuals in the previously defined 3-dose mRNA-1273 cohort study population with one eligible 2-dose mRNA-1273 individual. The date of study enrollment (and date at which inclusion/exclusion criteria were evaluated) for both the 3-dose recipient and their 2-dose match was 7 days after the third dose of the 3-dose recipient. Booster recipients with no valid 2-dose match were dropped from the study. The derivation of the BNT162b2 and mRNA-1273 cohorts is illustrated in Fig. 1, and the demographic and clinical characteristics of the cohorts are shown in Table S1 (Supplementary Material).

Booster effectiveness was assessed by comparing the cumulative SARS-CoV-2 incidence proportion between 2-dose and 3-dose recipients for each vaccine. In this study, incidence is defined as any positive SARS-CoV-2 PCR test, including tests that were designated as “symptomatic” or “asymptomatic” by the ordering provider. In a secondary analysis, we considered only positive tests that were designated as symptomatic (see Figure S1, Supplementary Material). Cumulative SARS-CoV-2 incidence proportion was estimated via a Kaplan–Meier analysis. The Kaplan–Meier curve was fitted using the *KaplanMeierFitter* module from the *lifelines* survival analysis library (version 0.26.3) in Python (Version 3.9.5, www.python.org). The cumulative incidence at time *t* is the estimated proportion of patients who tested positive for SARS-CoV-2 on or before time *t* (i.e. 1 minus the standard Kaplan–Meier estimate). A two-sided log-rank test was used to compare the incidence distributions between the 2-dose and 3-dose cohorts and to calculate a *P*-value. Data was right censored at the end of the study period; individuals who did not test positive by the end of the study were considered as negative for SARS-CoV-2 infection.

We calculated the incidence rate ratio (IRR) of positive SARS-CoV-2 PCR tests between the 2-dose and 3-dose cohorts for the BNT162b2 and mRNA-1273 vaccines. Incidence rates were defined as the number of patients who tested positive for SARS-CoV-2 in the given time period divided by the total number of at-risk person-days contributed to that time period. Individuals contributed at-risk days from their date of study enrollment until they tested positive for SARS-CoV-2, died, or until the end of the study period (whichever came first). We report incidence rates per 100 person days. The IRR was calculated as the incidence rate of the 3-dose cohort divided by the incidence rate of the 2-dose cohort. In addition, 95% CI for the IRR estimates were calculated using the same methodology as described previously (39).

### Cohort disease severity analysis

We fit Kaplan–Meier curves for the 2-dose and 3-dose BNT162b2 and mRNA-1273 cohorts using the following outcomes indicative of severe COVID-19: (1) 14-day COVID-19 associated hospitalization (hospital admission within 14 days after a first positive SARS-CoV-2 PCR test); and (2) 14-day COVID-19 associated ED admission (ED admission within 14 days after a first positive SARS-CoV-2 PCR test). For each outcome, we report the cumulative incidence proportion over time from the date of study enrollment, and the IRR between the 2-dose and 3-dose cohorts. IRRs were calculated as described above.

### Test-negative study design

We performed a test-negative case-control study design to assess whether BNT162b2 and mRNA-1273 booster vaccination compensates for waning in VE over time. This study design mirrored that outlined in the previous durability analysis of the BNT162b2 vaccine and a study on intraseason waning effectiveness of influenza vaccination (40, 41). We used conditional logistic regression (CLR) to estimate the odds of symptomatic SARS-CoV-2 infection over time following vaccination for individuals who had received two vs. three doses, while adjusting for relevant covariates. Symptomatic infection was defined as a positive SARS-CoV-2 PCR test that was not designated as “asymptomatic” by the ordering provider (subsequently referred to as “symptomatic tests”) (42). For recipients of 2-dose and 3-dose regimens of BNT162b2 and mRNA-1273, we compared the odds of symptomatic infection for a range of time intervals after the initial vaccine sequence to the odds of symptomatic infection within the first 1 to 6 days after the first dose. VE was estimated as (1-Odds Ratio) $\times$ 100% (43).

Cases were defined as the first positive symptomatic test for a given individual. Subsequent positive tests for the same individual were not considered as a case. Controls were defined as negative symptomatic tests with no prior positive tests (asymptomatic or symptomatic) for the same individual. Individuals who met the inclusion and exclusion criteria described previously were eligible to contribute cases and controls from the date of their first vaccine dose until they (i) had any positive test result (symptomatic or asymptomatic), (ii) died, or (iii) reached the end of the study period. If an individual contributed a negative symptomatic test 15 or fewer days before a positive test result, that test was excluded as a possible false negative result. If an individual contributed multiple negative test results within 15 days of each other, then one of those negative tests was selected randomly as a control and the others were dropped. This step was used to avoid duplicate counting of controls from a single symptomatic illness. Additionally, if an individual contributed more than three eligible negative symptomatic tests over the study period, then three tests were randomly selected as controls while the others were dropped, as has been described in previous test-negative studies of COVID-19 vaccines (9, 39).

After the implementation of the inclusion and exclusion criteria as well as the subsampling of negative tests as described, the BNT162b2 test-negative study population included 74,570 tests from 59,685 unique individuals. Among these, there were 6,033 tests from 1-dose recipients (at the time of the test), 55,283 from 2-dose recipients, and 13,254 from 3-dose recipients (third dose at least 14 days before the test). The mRNA-1273 test-negative study population included 35,090 tests from 28,951 unique individuals. Among these, there were 3,661 tests from 1-dose recipients, 27,213 from 2-dose recipients, and 4,216 from 3-dose recipients (third dose at least 14 days before the test).

This analysis considered many of the same key risk factors used in the previously defined cohort study. The primary exposure of interest and each covariate, denoted as X_1_to X_9_ in the CLR equation, is described below. The demographic and clinical characteristics of the populations used in the BNT162b2 and mRNA-1273 test-negative analyses are shown in Tables S6 and S7 (Supplementary Material), respectively.

*Primary exposure:*

1.
${X_1}$ : time since dose 1 or dose 2, defined as the number of days between the symptomatic PCR test and the date of the first or second dose, respectively. This was split into the following groups:
2. Among tests taken when the individual had received only one prior vaccine dose: 0 to 6, 7 to 13, and 14 to 28 days since the first vaccine dose. The time period 0 to 6 days since the first dose was considered as the reference.
3. Among tests taken when the individual had received two prior vaccine doses: 0 to 6, 7 to 13, 14 to 73, 74 to 133, 135 to 193, 194 to 253, 254 to 313, and 314+ days since the second vaccine dose.
4. Among tests taken when the individual had received three prior vaccine doses (with the third dose at least 14 days prior to the test): 135 to 193, 194 to 253, 254 to 313, and 314+ days since the second vaccine dose. Note that only the time periods 135+ days were considered because third doses received fewer than 120 days since the second dose were considered off-protocol and full protective immunity from the third vaccine dose is estimated to begin 14 days following administration.

*Covariates:*

1.
${X_2}$ : age in years as of the study start date (2020 December 16), modeled as a linear spline with knots at 25, 35, 45, 55, 65, 75, and 85 years. The minimum age (18 years old) was considered the reference category.
2.
${X_3}$ : number of Elixhauser comorbidities in the past 5 years, bucketed into four groups (0, 1 to 4, 5 to 9, and 10+). A score of 0 was considered the reference category.
3.
${X_4}$ : race, categorized into seven groups (Asian, Black/African American, Native American, Native Hawaiian/Pacific Islander, other, White/Caucasian, and unknown). White/Caucasian was considered the reference category because it comprised the majority of the individuals in the study.
4.
${X_5}$ : ethnicity, categorized into three groups (Hispanic/Latino, not Hispanic/Latino, and unknown). Not Hispanic/Latino was considered the reference category because it comprised most of the individuals in the study.
5.
${X_6}$ : sex, categorized into three groups (female, male, and unknown).
6.
${X_7}$ : immunosuppressant medication usage in the past 1 year, yes or no.

*Stratification factors:*

1.
${X_8}$ : county of primary residence for the individual.
2.
${X_9}$ : calendar week of the second dose, categorized in 1-week intervals.

For our outcome of interest (i.e. symptomatic SARS-CoV-2 infection), we fit a CLR model to estimate the odds of experiencing the outcome of interest in the defined time intervals after the date of the first and second vaccine doses, while adjusting for the covariates described above. The CLR model was defined by the equation,

$log( {\frac{{p\ Outcome}}{{1 - p\ Outcome}}} )\ = \ {\beta _0} + \ {\beta _1}{X_1} + \ {\beta _2}{X_2} + {\beta _3}{X_3}\ + {\beta _4}{X_4} + {\beta _5}{X_5}\ + {\beta _6}{X_6} + {\beta _7}{X_7} + Strata[{X_8},\ {X_9}$ ], where the covariates and the strata variables ${X_1} - {X_9}$ are described in the section above.

The model was fit using the *clogit* function from the *survival* package (version 3.2.11) in R (version, 4.1.0, www.r-project.org, Vienna, Austria). CIs and tests were based upon the Wald method. Odds ratios were considered statistically significant if the confidence intervals did not include 1.

## Authors’ Contributions

M.N, R.M, A.P, J.O., P.L, A.V, and V.S designed the research; R.M, A.P, J.O, and C.P performed the research; all authors analyzed the data; A.B and V.S contributed analytic tools; and all authors wrote the manuscript.

## Supplementary Material

pgac042_Supplemental_Files

## Data Availability

After publication, the data will be made available upon reasonable requests to the corresponding author. A proposal with a detailed description of study objectives and the statistical analysis plan will be needed for evaluation of the reasonability of requests. Deidentified data will be provided after approval from the corresponding author and the Mayo Clinic.

## Notes

**Competing Interest:** M.N., R.M., A.P., C.P., P.L., A.V., and V.S. are employees of nference and have financial interests in the company. nference is collaborating with biopharmaceutical companies on data science initiatives unrelated to this study. These collaborations had no role in study design, data collection and analysis, decision to publish, or preparation of the manuscript. Mayo Clinic holds equity in the company sponsoring the analysis presented in this manuscript. J.O. has received small grants from nference, Inc., and personal consulting fees from Bates College and Elsevier Inc. All of these activities are outside of the present work. M.D.S. receives research funding for the HERO-Together Vaccine SE Registry from Pfizer via Duke University. A.V. reports being an inventor for Mayo Clinic Travel App interaction with Smart Medical Kit and Medical Kit for Pilgrims. A.B. is supported by grants from NIAID (grants AI110173 and AI120698), Amfar (#109593), and Mayo Clinic (HH Shieck Khalifa Bib Zayed Al-Nahyan Named Professorship of Infectious Diseases). A.B. is a paid consultant for Abbvie, Gilead, Freedom Tunnel, Pinetree therapeutics Primmune, Immunome, MarPam, and Flambeau Diagnostics, is a paid member of the DSMB for Corvus Pharmaceuticals, Equilium, and Excision Biotherapeutics, has received fees for speaking for Reach MD and Medscape, owns equity for scientific advisory work in Zentalis and nference, and is the founder and President of Splissen therapeutics. D.C., C.V., J.G., H.G., and L.S. have no interests to disclose. This research has been reviewed by the Mayo Clinic Conflict of Interest Review Board and is being conducted in compliance with Mayo Clinic Conflict of Interest policies.
