# Supplementary material for: Third dose vaccination with mRNA-1273 or BNT162b2 vaccines improves protection against SARS-CoV-2 infection
Source: PNAS Nexus. 2022 Apr 28;1(2):pgac042. doi: 10.1093/pnasnexus/pgac042 (PMC9802350; doi:10.1093/pnasnexus/pgac042)
Supplement: pgac042_Supplemental_Files [file pgac042_supplemental_files.docx]

**Supplementary material for:**

### **Third dose vaccination with mRNA-1273 or BNT162b2 vaccines improves protection against SARS-CoV-2 infection**

Michiel J.M. Niesen,^1,+^ Robert Matson,^1,+^ Arjun Puranik,^1,+^ John C. O’Horo,^2,3,+^ Colin Pawlowski,^1^ Celine Vachon,^4^ Douglas Challener,^2^ Abinash Virk,^2^ Melanie Swift,^5^ Leigh Speicher,^6^ Joel Gordon,^7^ Holly Geyer,^8^ Patrick J. Lenehan,^1^ A.J. Venkatakrishnan,^1^ Venky Soundararajan,^1,*^ Andrew Badley^2,9,*^

^1^nference, Cambridge, Massachusetts 02139, USA

^2^Division of Infectious Diseases, Mayo Clinic, Rochester, Minnesota 55905, USA

^3^Division of Pulmonary and Critical Care Medicine, Mayo Clinic, Rochester, Minnesota 55905, USA

^4^Division of Quantitative Health Science, Mayo Clinic, Rochester, Minnesota, 55905, USA

^5^Division of Preventive, Occupational and Aerospace Medicine, Mayo Clinic, Rochester, Minnesota 55902, USA

^6^Division of General Internal Medicine, Mayo Clinic, Jacksonville, Florida, 32224 55905

^7^Department of Family Medicine, Mayo Clinic Health System, Mankato, Minnesota, 56001

^8^Division of Hospital Internal Medicine, Mayo Clinic, Phoenix, Arizona, 85054

^9^Department of Molecular Medicine, Mayo Clinic, Rochester, Minnesota 55905, USA

^+^Equal contributions

^*^Correspondence: [venky@nference.net](mailto:venky@nference.net), [Badley.Andrew@mayo.edu](mailto:Badley.Andrew@mayo.edu)

**Index**

- **Figure S1**: Kaplan-Meier analysis of the relative risk of symptomatic SARS-CoV-2 infection.
- **Figure S2**: Kaplan-Meier analysis of the relative risk of SARS-CoV-2 infection for immunocompromised individuals.
- **Figure S3**: Kaplan-Meier analysis of the relative risk of SARS-CoV-2 infection for individuals 50 years of age and above.
- **Figure S4**: Kaplan-Meier analysis of the relative risk of SARS-CoV-2 infection for individuals 18-49 years of age.
- **Figure S5**: Kaplan-Meier analysis of the relative risk of SARS-CoV-2 infection and subsequent emergency department admission.
- **Figure S6**: Distribution of time between doses.
- **Table S1**: Demographic and tracked comorbidities of the study participants.
- **Table S2**: SARS-CoV-2 Incidence rates in the 3-dose BNT162b2 cohort and the 1:1 matched 2-dose cohort.
- **Table S3**: SARS-CoV-2 Incidence rates in the 3-dose mRNA-1273 cohort and the 1:1 matched 2-dose cohort.
- **Table S4**: SARS-CoV-2 test outcomes and odds ratio of positive versus negative test outcomes compared to time since 2nd vaccine dose with BNT162b2.
- **Table S5**: SARS-CoV-2 test outcomes and odds ratio of positive versus negative test outcomes compared to time since 2nd vaccine dose with mRNA-1273
- **Table S6**: Clinical characteristics of population used in test-negative analysis for BNT162b2.
- **Table S7**: Clinical characteristics of population used in test-negative analysis for mRNA-1273.


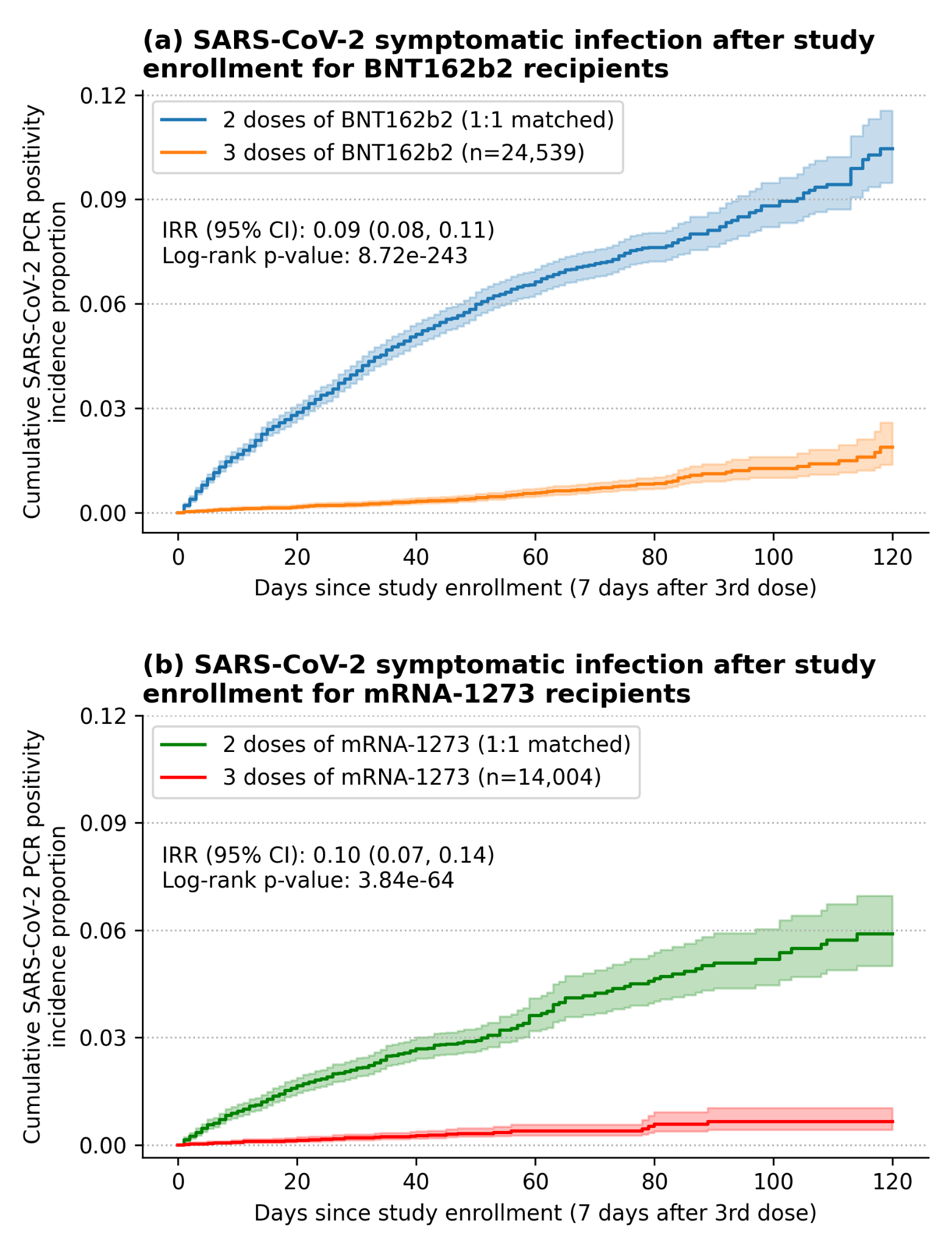


**Figure S1: Kaplan-Meier analysis of the relative risk of symptomatic SARS-CoV-2 infection.** Symptomatic SARS-CoV-2 infection is defined as a positive PCR test accompanied by one or more symptoms for COVID-19. Shown is the cumulative incidence of symptomatic SARS-CoV-2 infections for: (**a**) 3-dose BNT162b2 recipients (orange) and the 1:1 matched 2-dose cohort (blue), and (**b**) 3-dose mRNA-1273 recipients (red) and the 1:1 matched 2-dose cohort (green). Shaded regions correspond to 95% confidence intervals.


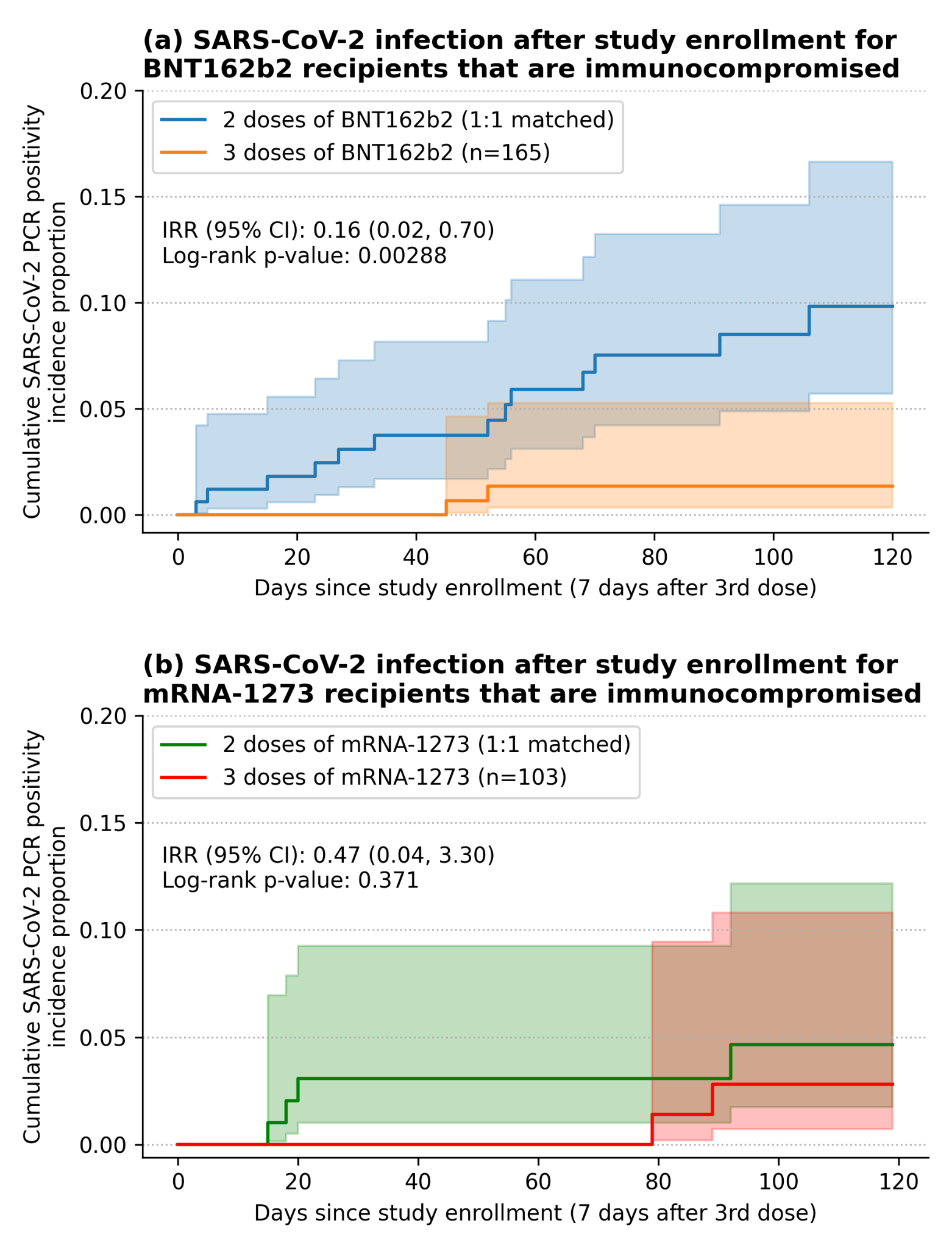


**Figure S2: Kaplan-Meier analysis of the relative risk of SARS-CoV-2 infection for immunocompromised individuals.** Shown are the cumulative incidence rates of positive SARS-CoV-2 PCR tests for: (**a**) 3-dose BNT162b2 immunocompromised recipients (orange) and the matched 2-dose cohort (blue), and (**b**) 3-dose immunocompromised mRNA-1273 recipients (red) and their 1:1 matched 2-dose cohort (green). Shaded regions correspond to 95% confidence intervals. Immunocompromised individuals are defined here as those with known immunosuppressant drug prescription within 1 year of their 2^nd^ vaccine dose.


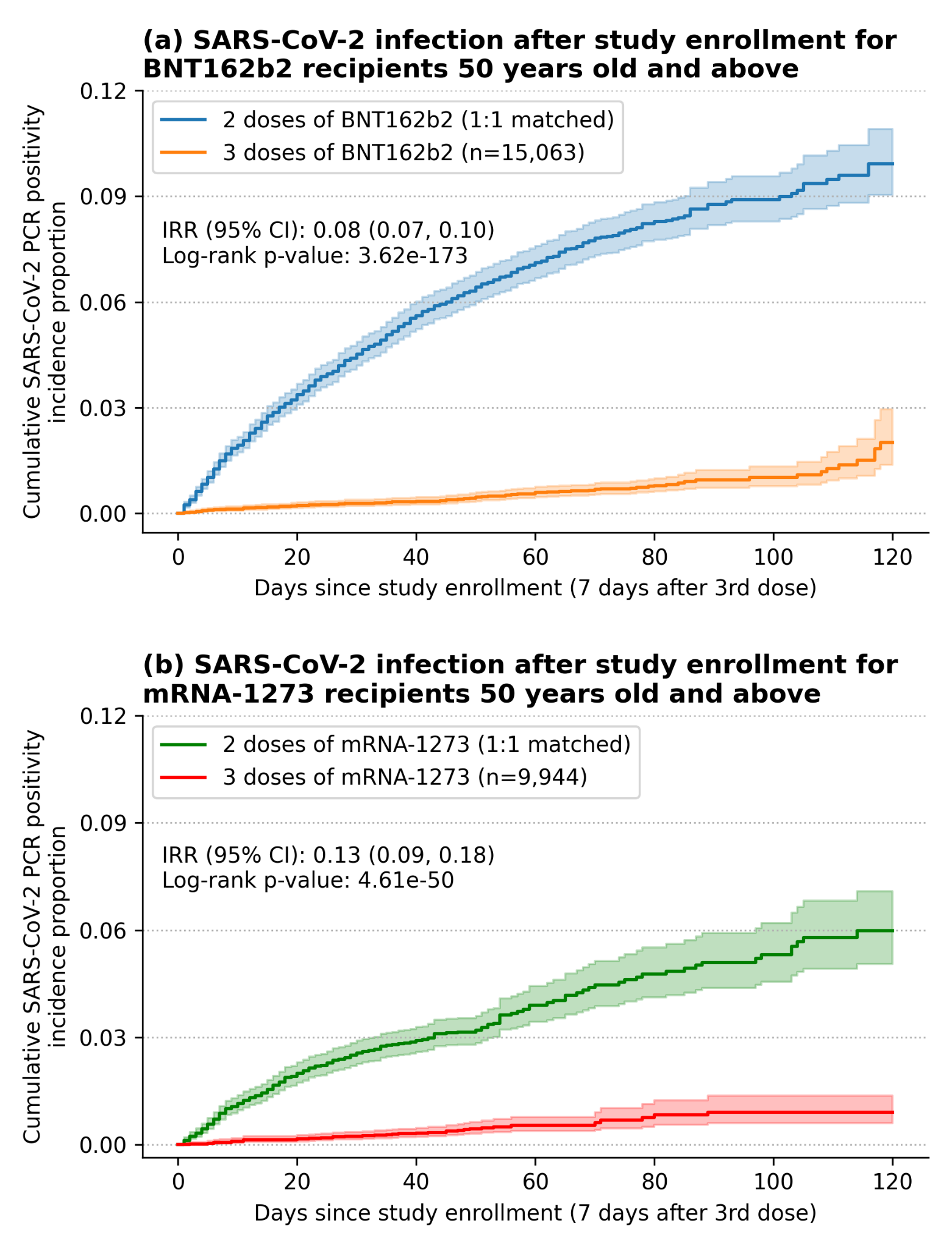


**Figure S3: Kaplan-Meier analysis of the relative risk of SARS-CoV-2 infection for individuals 50 years of age and above.** For this subpopulation, shown are the cumulative incidence rates of positive SARS-CoV-2 PCR tests for: (**a**) 3-dose BNT162b2 recipients (orange) and their 1:1 matched 2-dose cohort (blue), and (**b**) 3-dose mRNA-1273 recipients (red) and their 1:1 matched 2-dose cohort (green). Shaded regions correspond to 95% confidence intervals.


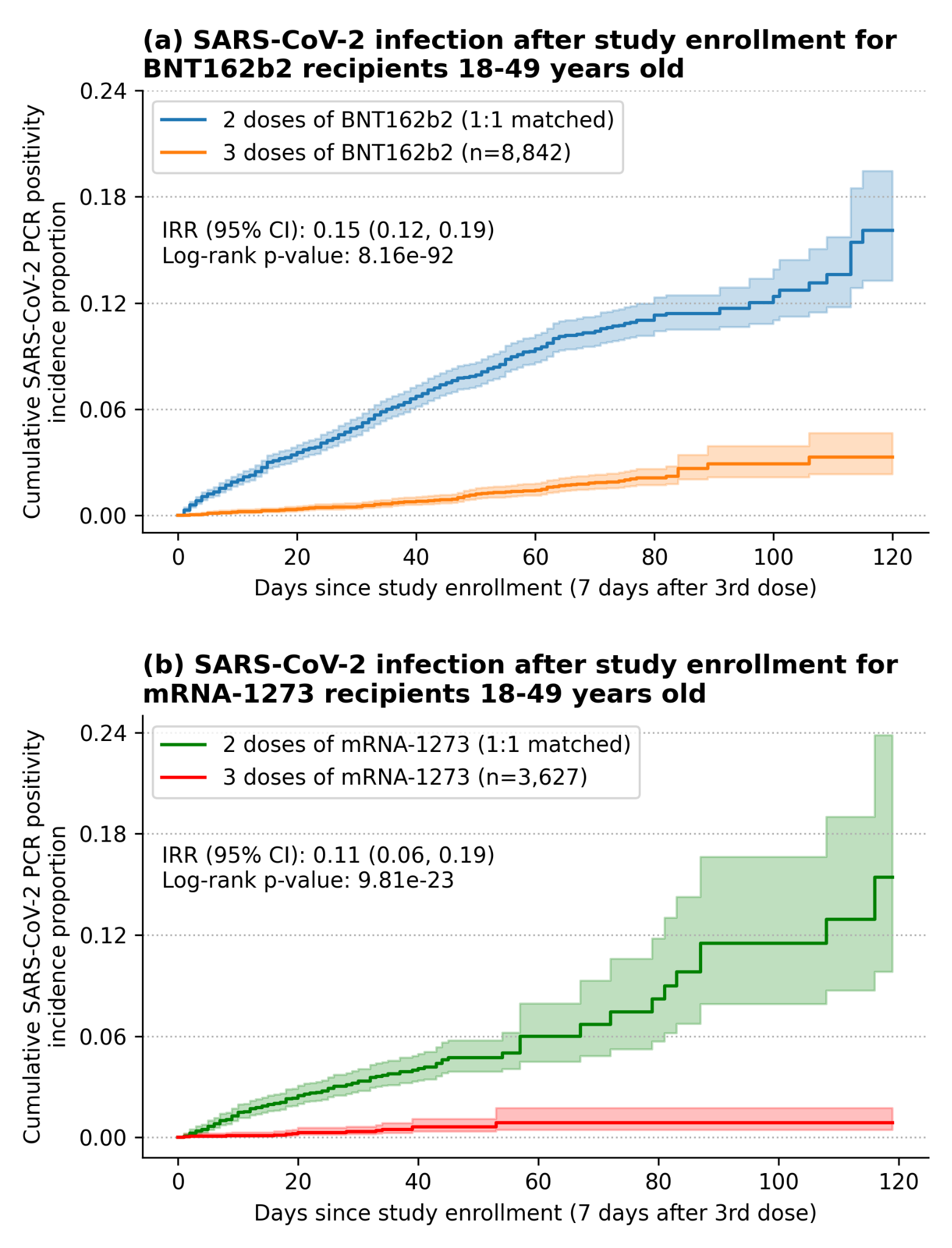


**Figure S4: Kaplan-Meier analysis of the relative risk of SARS-CoV-2 infection for individuals 18-49 years of age.** For this subpopulation, shown are the cumulative incidence rates of positive SARS-CoV-2 PCR tests for: (**a**) 3-dose BNT162b2 recipients (orange) and their 1:1 matched 2-dose cohort (blue), and (**b**) 3-dose mRNA-1273 recipients (red) and their 1:1 matched 2-dose cohort (green). Shaded regions correspond to 95% confidence intervals.


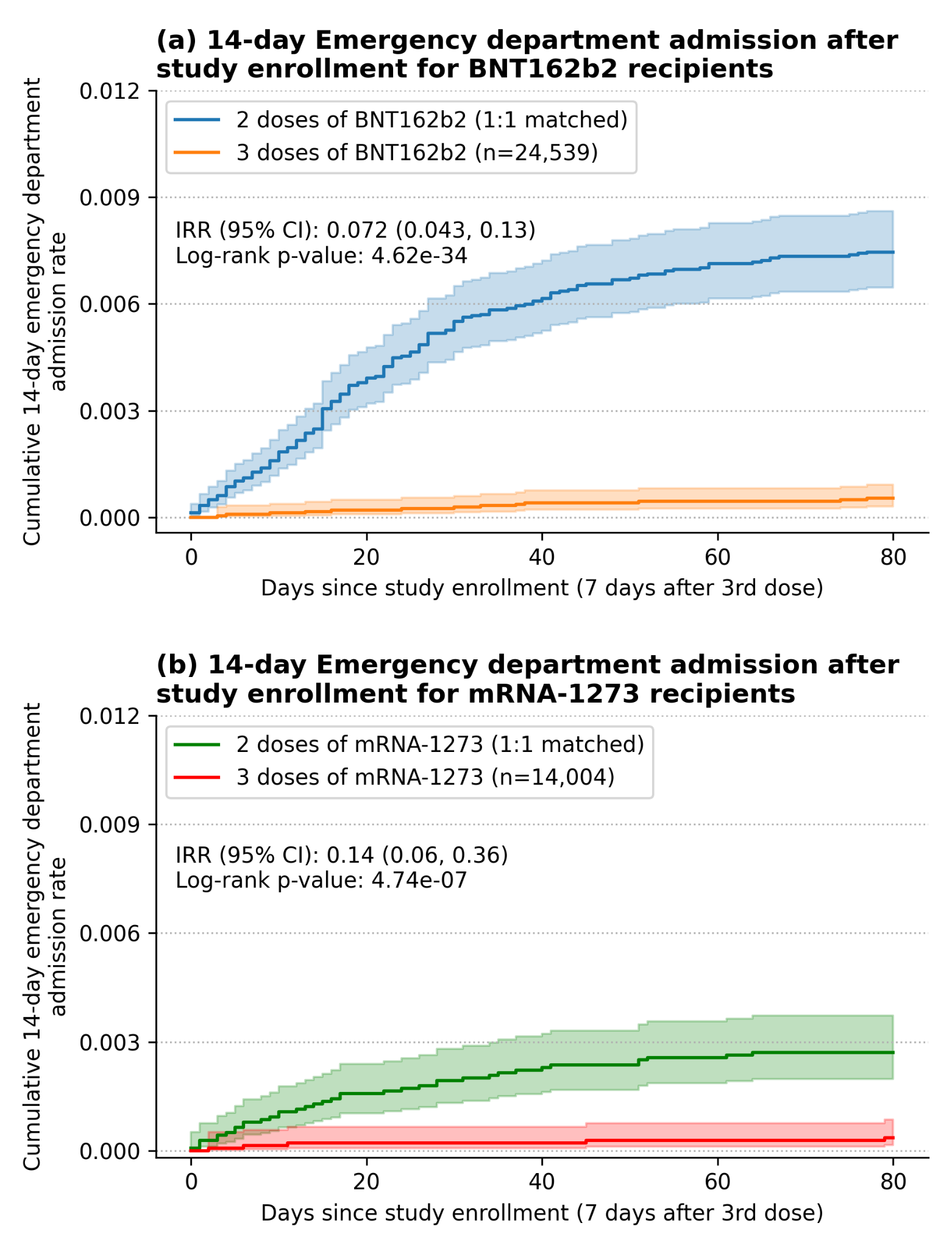


**Figure S5: Kaplan-Meier analysis of the relative risk of SARS-CoV-2 infection and subsequent emergency department admission.** SARS-CoV-2 infection and subsequent emergency department (ED) admission is defined as a positive PCR test after study enrollment along with ED admission within 14 days. Shown are the cumulative rates for: (**a**) 3-dose BNT162b2 recipients (orange) and their 1:1 matched 2-dose cohort (blue), and (**b**) 3-dose mRNA-1273 recipients (red) and their 1:1 matched 2-dose cohort (green). Shaded regions correspond to 95% confidence intervals.

**
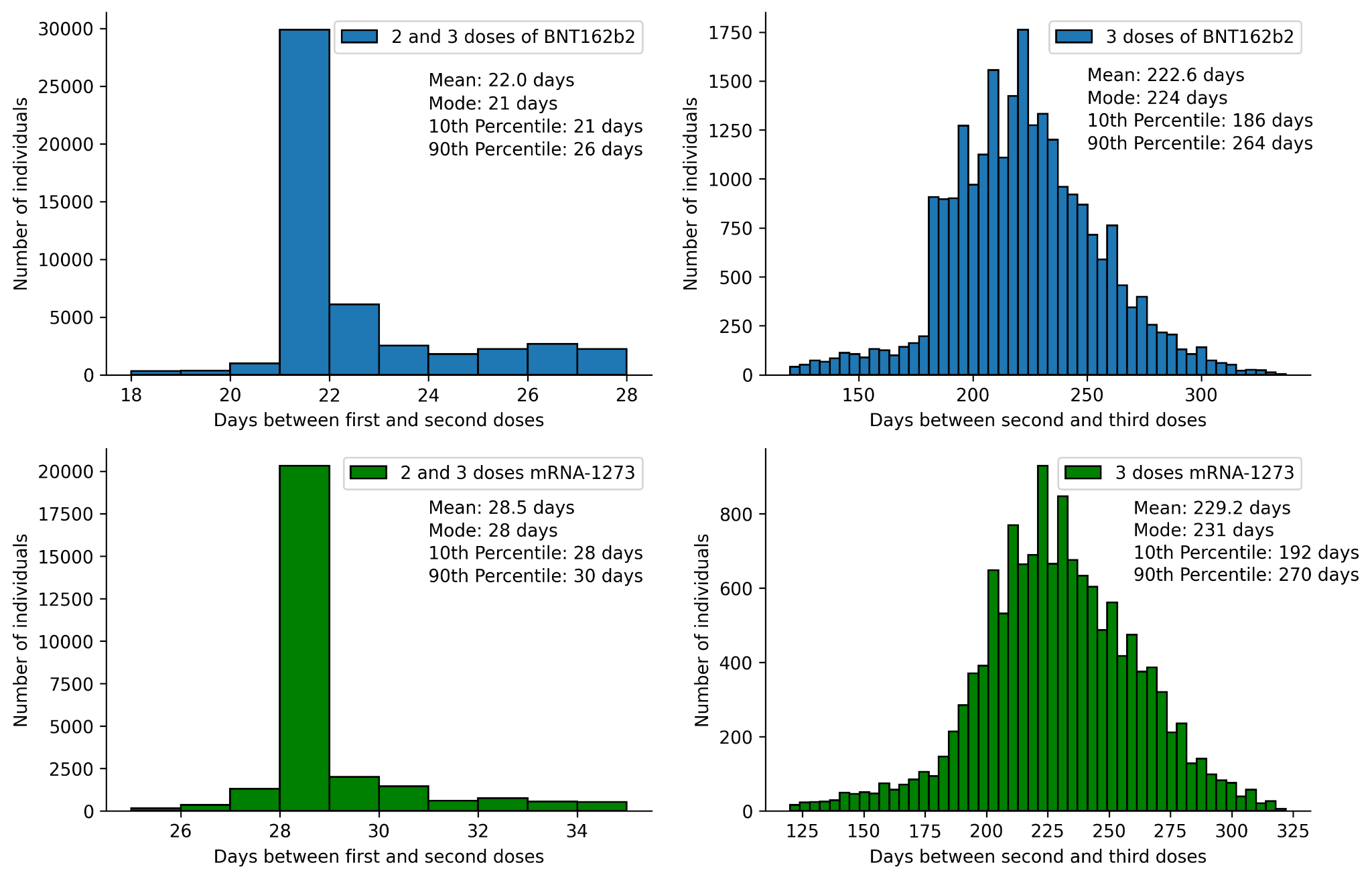
**

**Figure S6: Distribution of time between doses.** (**left**) Counts of the time between 1st and 2nd dose for all study participants included in the cohort analysis. (**right**) Counts of the time between second and third dose for all 3-dose recipients included in the cohort analysis. Data shown separately for BNT162b2 (blue) and mRNA-1273 (green) recipients.

**Table S1: Demographic and tracked comorbidities of the study participants.**

|  | **Individuals, No. (%)** |  |  | **Individuals, No. (%)** |  |  |
| --- | --- | --- | --- | --- | --- | --- |
| **Clinical covariate** | **2-dose BNT162b2 cohort (n=24,539)** | **3-dose BNT162b2 cohort (n=24,539)** | **Cohen’s D** | **2-dose mRNA-1273 cohort (n=14,004)** | **3-dose mRNA-1273 cohort (n=14,004)** | **Cohen’s D** |
| **Sex:**   - Female | 14,031 (57.2%) | 14,031 (57.2%) | 0.00 | 7,638 (54.5%) | 7,638 (54.5%) | 0.00 |
| - Male | 10,507 (42.8%) | 10,507 (42.8%) | 0.00 | 6,366 (45.5%) | 6,366 (45.5%) | 0.00 |
| - Unknown | 1 (0.0%) | 1 (0.0%) | 0.00 | 0 (0.0%) | 0 (0.0%) | N/A |
| **Age (in years):**   - 18-24 | 935 (3.8%) | 935 (3.8%) | 0.17 | 382 (2.7%) | 382 (2.7%) | 0.10 |
| - 25-34 | 3,049 (12.4%) | 3,049 (12.4%) | 0.12 | 1,080 (7.7%) | 1,080 (7.7%) | 0.23 |
| - 35-44 | 3,464 (14.1%) | 3,464 (14.1%) | 0.09 | 1,554 (11.1%) | 1,554 (11.1%) | 0.06 |
| - 45-54 | 3,934 (16.0%) | 3,934 (16.0%) | 0.06 | 1,900 (13.6%) | 1,900 (13.6%) | 0.13 |
| - 55-64 | 5,986 (24.4%) | 5,986 (24.4%) | 0.12 | 3,221 (23.0%) | 3,221 (23.0%) | 0.16 |
| - 65-74 | 4,311 (17.6%) | 4,311 (17.6%) | 0.04 | 3,840 (27.4%) | 3,840 (27.4%) | 0.06 |
| - 75-84 | 2,257 (9.2%) | 2,257 (9.2%) | 0.10 | 1,534 (11.0%) | 1,534 (11.0%) | 0.12 |
| - 85+ | 603 (2.5%) | 603 (2.5%) | 0.16 | 493 (3.5%) | 493 (3.5%) | 0.27 |
| **Race:**   - Asian | 530 (2.2%) | 530 (2.2%) | 0.00 | 131 (0.9%) | 131 (0.9%) | 0.00 |
| - Black / African American | 279 (1.1%) | 279 (1.1%) | 0.00 | 124 (0.9%) | 124 (0.9%) | 0.00 |
| - Native American | 3 (0.0%) | 3 (0.0%) | 0.00 | 3 (0.0%) | 3 (0.0%) | 0.00 |
| - Other | 137 (0.6%) | 137 (0.6%) | 0.00 | 23 (0.2%) | 23 (0.2%) | 0.00 |
| - Unknown | 373 (1.5%) | 373 (1.5%) | 0.00 | 241 (1.7%) | 241 (1.7%) | 0.00 |
| - White / Caucasian | 23,217 (94.6%) | 23,217 (94.6%) | 0.00 | 13,482 (96.3%) | 13,482 (96.3%) | 0.00 |
| **Ethnicity:**   - Hispanic or Latino | 304 (1.2%) | 304 (1.2%) | 0.00 | 110 (0.8%) | 110 (0.8%) | 0.00 |
| - Not Hispanic or Latino | 23,780 (96.9%) | 23,780 (96.9%) | 0.00 | 13,604 (97.1%) | 13,604 (97.1%) | 0.00 |
| - Unknown | 455 (1.9%) | 455 (1.9%) | 0.00 | 290 (2.1%) | 290 (2.1%) | 0.00 |
| **State of primary residence:**   - Arizona | 4,783 (19.5%) | 4,783 (19.5%) | 0.00 | 1,481 (10.6%) | 1,481 (10.6%) | 0.00 |
| - Florida | 3,356 (13.7%) | 3,356 (13.7%) | 0.00 | 2,853 (20.4%) | 2,853 (20.4%) | 0.00 |
| - Iowa | 5,415 (22.1%) | 5,415 (22.1%) | 0.00 | 2,159 (15.4%) | 2,159 (15.4%) | 0.00 |
| - Minnesota | 10,808 (44.0%) | 10,808 (44.0%) | 0.00 | 7,443 (53.1%) | 7,443 (53.1%) | 0.00 |
| - Other | 177 (0.7%) | 177 (0.7%) | 0.00 | 68 (0.5%) | 68 (0.5%) | 0.00 |
| **Number of Elixhauser comorbidities:**   - 0 | 20,433 (83.3%) | 20,433 (83.3%) | 0.00 | 11,353 (81.1%) | 11,353 (81.1%) | 0.00 |
| - 1-4 | 2,520 (10.3%) | 2,520 (10.3%) | 0.01 | 1,519 (10.8%) | 1,519 (10.8%) | 0.02 |
| - 5-9 | 1,249 (5.1%) | 1,249 (5.1%) | 0.02 | 867 (6.2%) | 867 (6.2%) | 0.04 |
| - 10+ | 337 (1.4%) | 337 (1.4%) | 0.03 | 265 (1.9%) | 265 (1.9%) | 0.05 |
| **Immunocompromised:**   - No | 24,373 (99.3%) | 24,373 (99.3%) | 0.00 | 13,900 (99.3%) | 13,900 (99.3%) | 0.00 |
| - Yes | 166 (0.7%) | 166 (0.7%) | 0.00 | 104 (0.7%) | 104 (0.7%) | 0.00 |
| **Previous PCR tests:**   - 0 | 11,883 (48.4%) | 11,883 (48.4%) | 0.00 | 7,535 (53.8%) | 7,535 (53.8%) | 0.00 |
| - 1 | 5,671 (23.1%) | 5,671 (23.1%) | 0.00 | 3,179 (22.7%) | 3,179 (22.7%) | 0.00 |
| - 2 | 2,809 (11.4%) | 2,809 (11.4%) | 0.00 | 1,503 (10.7%) | 1,503 (10.7%) | 0.00 |
| - 3 | 1,523 (6.2%) | 1,523 (6.2%) | 0.00 | 675 (4.8%) | 675 (4.8%) | 0.00 |
| - 4 | 798 (3.3%) | 798 (3.3%) | 0.00 | 332 (2.4%) | 332 (2.4%) | 0.00 |
| - 5+ | 1,855 (7.6%) | 1,855 (7.6%) | 0.02 | 780 (5.6%) | 780 (5.6%) | 0.04 |
| **Calendar month of second dose:**   - 2021-01 | 3,084 (12.6%) | 3,138 (12.8%) | 0.01 | 336 (2.4%) | 343 (2.4%) | 0.00 |
| - 2021-02 | 5,772 (23.5%) | 5,678 (23.1%) | 0.01 | 3,022 (21.6%) | 3,008 (21.5%) | 0.00 |
| - 2021-03 | 5,243 (21.4%) | 5,443 (22.2%) | 0.02 | 5,092 (36.4%) | 5,167 (36.9%) | 0.01 |
| - 2021-04 | 8,694 (35.4%) | 8,595 (35.0%) | 0.01 | 4,705 (33.6%) | 4,638 (33.1%) | 0.01 |
| - 2021-05 | 1,672 (6.8%) | 1,616 (6.6%) | 0.01 | 836 (6.0%) | 837 (6.0%) | 0.00 |
| - 2021-06 | 72 (0.3%) | 68 (0.3%) | 0.00 | 13 (0.1%) | 11 (0.1%) | 0.00 |
| - 2021-07 | 2 (0.0%) | 1 (0.0%) | 0.01 | 0 (0.0%) | 0 (0.0%) | N/A |

**Table S2: SARS-CoV-2 Incidence rates in the 3-dose BNT162b2 cohort and the 1:1 matched 2-dose cohort.**

| **Time period** | **3-dose BNT162b2 COVID-19 Incidence (per 100 person days)** | **2-dose BNT162b2 COVID-19 Incidence (per 100 person days)** | **Incidence rate ratio (95% CI)** |
| --- | --- | --- | --- |
| Days 1 to 14 | 0.0155 | 0.206 | 0.07 (0.05, 0.10) |
| Days 15 to 28 | 0.0106 | 0.155 | 0.07 (0.04, 0.10) |
| Days 29 to 42 | 0.0119 | 0.147 | 0.08 (0.05, 0.12) |
| Days 43 to 56 | 0.0206 | 0.111 | 0.19 (0.13, 0.27) |
| Day 1 onwards | 0.0152 | 0.138 | 0.11 (0.09, 0.13) |
| Day 8 onwards | 0.0149 | 0.125 | 0.12 (0.10, 0.14) |
| Day 15 onwards | 0.0154 | 0.117 | 0.13 (0.11, 0.16) |
| Day 22 onwards | 0.0165 | 0.114 | 0.14 (0.12, 0.17) |

**Table S3: SARS-CoV-2 Incidence rates in the 3-dose mRNA-1273 cohort and the 1:1 matched 2-dose cohort.**

| **Time period** | **3-dose mRNA-1273 COVID-19 Incidence (per 100 person days)** | **2-dose mRNA-1273 COVID-19 Incidence (per 100 person days)** | **Incidence rate ratio (95% CI)** |
| --- | --- | --- | --- |
| Days 1 to 14 | 0.0107 | 0.121 | 0.09 (0.05, 0.14) |
| Days 15 to 28 | 0.00992 | 0.082 | 0.12 (0.07, 0.21) |
| Days 29 to 42 | 0.00832 | 0.0605 | 0.14 (0.06, 0.28) |
| Days 43 to 56 | 0.00844 | 0.0444 | 0.19 (0.06, 0.51) |
| Day 1 onwards | 0.00911 | 0.0793 | 0.11 (0.08, 0.15) |
| Day 8 onwards | 0.00868 | 0.0684 | 0.13 (0.09, 0.17) |
| Day 15 onwards | 0.00871 | 0.063 | 0.14 (0.09, 0.20) |
| Day 22 onwards | 0.00884 | 0.0549 | 0.16 (0.10, 0.24) |

**Table S4: SARS-CoV-2 test outcomes and odds ratio of positive versus negative test outcomes compared to time since 2nd vaccine dose with BNT162b2 (correspond with Figure 4).**

|  | **Time period** | **Symptomatic positive tests** | **Symptomatic negative tests** | **Crude odds ratio** | ***Adjusted* Odds ratio (95% CI)** | **Vaccine Effectiveness (95% CI)** |
| --- | --- | --- | --- | --- | --- | --- |
| **1 dose** | 1-6 days after first dose  *[baseline]* | 327 | 1343 | *1* | *1* |  |
|  | 7-13 days after first dose | 442 | 1633 | 1.11 (0.947, 1.3) | 1.12 (0.964, 1.31) | -12.0% (-31.0%, 3.6%) |
|  | 14-28 days after first dose | 276 | 2012 | 0.563 (0.473, 0.671) | 0.595 (0.501, 0.707) | 40.5% (29.3%, 49.9%) |
| **2 doses, no booster** | 0-6 days after second dose | 83 | 1013 | 0.337 (0.261, 0.434) | 0.424 (0.329, 0.547) | 57.6% (45.3%, 67.1%) |
|  | 7-13 days after second dose | 42 | 967 | 0.178 (0.128, 0.249) | 0.221 (0.158, 0.309) | 77.9% (69.1%, 84.2%) |
|  | 14-73 days after second dose | 285 | 7098 | 0.165 (0.139, 0.195) | 0.194 (0.163, 0.231) | 80.6% (76.9%, 83.7%) |
|  | 74-133 days after second dose | 1025 | 9354 | 0.45 (0.392, 0.516) | 0.363 (0.313, 0.42) | 63.7% (58.0%, 68.7%) |
|  | 135-193 days after second dose | 2331 | 13993 | 0.684 (0.602, 0.778) | 0.457 (0.397, 0.525) | 54.3% (47.5%, 60.3%) |
|  | 194-253 days after second dose | 2822 | 11890 | 0.975 (0.858, 1.11) | 0.527 (0.458, 0.606) | 47.3% (39.4%, 54.2%) |
|  | 254-313 days after second dose | 921 | 2991 | 1.26 (1.1, 1.46) | 0.529 (0.454, 0.617) | 47.1% (38.3%, 54.6%) |
|  | 314-355 days after second dose | 136 | 332 | 1.68 (1.33, 2.12) | 0.567 (0.453, 0.712) | 43.3% (28.8%, 54.7%) |
| **Boosted** | 135-193 days after second dose | 15 | 280 | 0.22 (0.129, 0.375) | 0.128 (0.0752, 0.217) | 87.2% (78.3%, 92.5%) |
|  | 194-253 days after second dose | 179 | 3561 | 0.206 (0.17, 0.25) | 0.0902 (0.0737, 0.111) | 91.0% (88.9%, 92.6%) |
|  | 254-313 days after second dose | 326 | 6815 | 0.196 (0.167, 0.232) | 0.0798 (0.0667, 0.0955) | 92.0% (90.5%, 93.3%) |
|  | 314-355 days after second dose | 137 | 1941 | 0.29 (0.235, 0.358) | 0.104 (0.0833, 0.131) | 89.6% (86.9%, 91.7%) |

**Table S5: SARS-CoV-2 test outcomes and odds ratio of positive versus negative test outcomes compared to time since 2nd vaccine dose with mRNA-1273 (correspond with Figure 4).**

|  | **Time period** | **Symptomatic positive tests** | **Symptomatic negative tests** | **Crude odds ratio** | ***Adjusted* Odds ratio (95% CI)** | **Vaccine Effectiveness (95% CI)** |
| --- | --- | --- | --- | --- | --- | --- |
| **1 dose** | 1-6 days after first dose  *[baseline]* | 235 | 668 |  |  |  |
|  | 7-13 days after first dose | 203 | 814 | 0.709 (0.572, 0.878) | 0.78 (0.633, 0.963) | 22.0% (3.7%, 36.7%) |
|  | 14-28 days after first dose | 145 | 1596 | 0.258 (0.206, 0.324) | 0.315 (0.25, 0.397) | 68.5% (60.3%, 75.0%) |
| **2 doses, no booster** | 0-6 days after second dose | 20 | 475 | 0.12 (0.0747, 0.192) | 0.168 (0.104, 0.272) | 83.2% (72.8%, 89.6%) |
|  | 7-13 days after second dose | 6 | 410 | 0.0416 (0.0183, 0.0944) | 0.0519 (0.0224, 0.12) | 94.8% (88.0%, 97.8%) |
|  | 14-73 days after second dose | 75 | 2404 | 0.0887 (0.0674, 0.117) | 0.0984 (0.0732, 0.132) | 90.2% (86.8%, 92.7%) |
|  | 74-133 days after second dose | 304 | 3707 | 0.233 (0.193, 0.282) | 0.209 (0.166, 0.263) | 79.1% (73.7%, 83.4%) |
|  | 135-193 days after second dose | 1094 | 8390 | 0.371 (0.315, 0.436) | 0.274 (0.223, 0.337) | 72.6% (66.3%, 77.7%) |
|  | 194-253 days after second dose | 1274 | 6798 | 0.533 (0.454, 0.625) | 0.342 (0.278, 0.421) | 65.8% (57.9%, 72.2%) |
|  | 254-313 days after second dose | 418 | 1708 | 0.696 (0.579, 0.835) | 0.368 (0.293, 0.463) | 63.2% (53.7%, 70.7%) |
|  | 314-339 days after second dose | 28 | 102 | 0.78 (0.501, 1.22) | 0.277 (0.177, 0.434) | 72.3% (56.6%, 82.3%) |
| **Boosted** | 135-193 days after second dose | 5 | 126 | 0.113 (0.0456, 0.279) | 0.0759 (0.0306, 0.188) | 92.4% (81.2%, 96.9%) |
|  | 194-253 days after second dose | 63 | 1506 | 0.119 (0.0887, 0.159) | 0.063 (0.0455, 0.0871) | 93.7% (91.3%, 95.5%) |
|  | 254-313 days after second dose | 94 | 2104 | 0.127 (0.0985, 0.164) | 0.0688 (0.0511, 0.0926) | 93.1% (90.7%, 94.9%) |
|  | 314-339 days after second dose | 31 | 287 | 0.307 (0.206, 0.458) | 0.109 (0.0696, 0.171) | 89.1% (82.9%, 93.0%) |

**Table S6: Clinical characteristics of population used in test-negative analysis for BNT162b2.**

| **Characteristic** | **Test-Negative controls**  1+ dose, on-protocol at time of negative symptomatic test | **Test-Negative cases**  1+ dose, on-protocol at time of positive symptomatic test | **Test-Negative boosted controls**  Third dose at least 14 days prior to negative symptomatic test, on-protocol at time test | **Test-Negative boosted cases**  Third dose at least 14 days prior to positive symptomatic test, on-protocol at time test |
| --- | --- | --- | --- | --- |
| Number of tests (in analyzable strata) | 65,223 | 9,347 | 12,597 | 657 |
| Number of individuals contributing tests (in analyzable strata) | 51,956 | 9,347 | 11,568 | 657 |
| Age (years)   - Mean (sd) - 18-24 - 25-34 - 35-44 - 45-54 - 55-64 - 65-74 - 75-84 - 85+ | 52.3 (19.2)  3,676 (7.1%)  8,491 (16.3%)  8,598 (16.5%)  7,306 (14.1%)  9,002 (17.3%)  7,334 (14.1%)  5,359 (10.3%)  2,190 (4.2%) | 50.7 (17.8)  584 (6.2%)  1,546 (16.5%)  1,828 (19.6%)  1,513 (16.2%)  1,731 (18.5%)  1,121 (12.0%)  758 (8.1%)  266 (2.8%) | 57.4 (18.7)  336 (2.9%)  1,517 (13.1%)  1,692 (14.6%)  1,384 (12.0%)  1,997 (17.3%)  2,282 (19.7%)  1,762 (15.2%)  598 (5.2%) | 54.9 (18.4)  29 (4.4%)  93 (14.2%)  100 (15.2%)  85 (12.9%)  121 (18.4%)  122 (18.6%)  87 (13.2%)  20 (3.0%) |
| State of Primary Residence   - Arizona - Florida - Iowa - Minnesota - Wisconsin - Other | 5,211 (10.0%)  6,653 (12.8%)  72 (0.1%)  26,240 (50.5%)  13,766 (26.5%)  14 (0.0%) | 740 (7.9%)  1,016 (10.9%)  53 (0.6%)  4,767 (51.0%)  2,762 (29.5%)  9 (0.1%) | 1,048 (9.1%)  1,081 (9.3%)  13 (0.1%)  6,532 (56.5%)  2,893 (25.0%)  1 (0.0%) | 55 (8.4%)  49 (7.5%)  400 (60.9%)  152 (23.1%)  1 (0.2%) |
| Sex   - Female - Male - Unknown | 31,973 (61.5%)  19,975 (38.4%)  8 (0.0%) | 5,465 (58.5%)  3,882 (41.5%) | 7,328 (63.3%)  4,238 (36.6%)  2 (0.0%) | 399 (60.7%)  258 (39.3%) |
| Race   - Asian - Black / African American - Native American - Native Hawaiian/Pacific Islander - White / Caucasian - Other - Unknown | 1,827 (3.5%)  1,401 (2.7%)  151 (0.3%)  51 (0.1%)  46,637 (89.8%)  1,202 (2.3%)  687 (1.3%) | 254 (2.7%)  216 (2.3%)  33 (0.4%) 12 (0.1%)  8,482 (90.7%)  222 (2.4%)  128 (1.4%) | 373 (3.2%)  160 (1.4%)  16 (0.1%)  7 (0.1%)  10,729 (92.7%)  198 (1.7%)  85 (0.7%) | 23 (3.5%)  13 (2.0%)  3 (0.5%)  0 (0%)   600 (91.3%)  10 (1.5%)  8 (1.2%) |
| Ethnicity   - Hispanic or Latino - Not Hispanic or Latino - Unknown | 2,102 (4.0%)  48,593 (93.5%)  1,261 (2.4%) | 376 (4.0%)  8,739 (93.5%)  232 (2.5%) | 339 (2.9%)  11,025 (95.3%)  204 (1.8%) | 20 (3.0%)  619 (94.2%)  18 (2.7%) |
| Number of Elixhauser comorbidities   - 0 - 1-4 - 5-9 - 10+ | 38,194 (73.5%)  2,221 (4.3%)  6,934 (13.3%)  4,607 (8.9%) | 7,054 (75.5%)  310 (3.3%)  1,311 (14.0%)  672 (7.2%) | 7,937 (68.6%)  631 (5.5%)  1,683 (14.5%)  1,317 (11.4%) | 463 (70.5%)  42 (6.4%)  90 (13.7%)  62 (9.4%) |
| Dates of second dose   - *Number with 2nd dose* - Earliest - 25th % - Median - 75th % - Latest | 60,298  2021-01-04  2021-02-10  2021-03-18  2021-04-20  2021-12-27 | 8,305  2021-01-05  2021-02-15  2021-03-30  2021-04-27  2021-12-27 | 12,597  2021-01-04  2021-01-28  2021-02-19  2021-03-23  2021-06-09 | 657  2021-01-07  2021-01-29  2021-02-24  2021-03-26  2021-05-17 |
| Time between second dose and test   - Minimum - 25th % - Median - 75th % - Maximum | 0  116  185  241  355 | 0  157  201  240  351 | 141  248  275  302  355 | 155  249  275  309  351 |
| Dates of third dose   - Earliest - 25th % - Median - 75th % - Latest |  |  | 2021-08-13  2021-09-27  2021-10-05  2021-10-20  2021-12-13 | 2021-08-14  2021-09-16  2021-10-04  2021-10-19  2021-12-13 |
| Time between third dose and test   - Minimum - 25th % - Median - 75th % - Maximum |  |  | 14  30  47  68  135 | 14  40  61  81  131 |

**Table S7: Clinical characteristics of population used in test-negative analysis for mRNA-1273.**

| **Characteristic** | **Test-Negative controls**  1+ dose, on-protocol at time of negative symptomatic test | **Test-Negative cases**  1+ dose, on-protocol at time of positive symptomatic test | **Test-Negative boosted controls**  Third dose at least 14 days prior to negative symptomatic test, on-protocol at time test | **Test-Negative boosted cases**  Third dose at least 14 days prior to positive symptomatic test, on-protocol at time test |
| --- | --- | --- | --- | --- |
| Number of tests (in analyzable strata) | 31,095 | 3,995 | 4,023 | 193 |
| Number of individuals contributing tests (in analyzable strata) | 25,561 | 3,995 | 3,798 | 193 |
| Age (years)   - Mean (sd) - 18-24 - 25-34 - 35-44 - 45-54 - 55-64 - 65-74 - 75-84 - 85+ | 55.9 (19.0)  1,615 (6.3%)  2,985 (11.7%)  3,358 (13.1%)  3,415 (13.4%)  4,635 (18.1%)  5,631 (22.0%)  2,525 (9.9%)  1,397 (5.5%) | 52.3 (17.9)  256 (6.4%)  538 (13.5%)  722 (18.1%)  626 (15.7%)  726 (18.2%)  719 (18.0%)  293 (7.3%)  115 (2.9%) | 62.4 (16.2)  73 (1.9%)  255 (6.7%)  307 (8.1%)  397 (10.5%)  747 (19.7%)  1,313 (34.6%)  499 (13.1%)  207 (5.5%) | 56.8 (17.6)  9 (4.7%)  27 (14.0%)  15 (7.8%)  25 (13.0%)  37 (19.2%)  51 (26.4%)  25 (13.0%)  4 (2.1%) |
| State of Primary Residence   - Arizona - Florida - Iowa - Minnesota - Wisconsin - Other | 1,603 (6.3%)  5,248 (20.5%)  50 (0.2%)  13,850 (54.2%)  4,805 (18.8%)  5 (0.0%) | 193 (4.8%)  657 (16.4%)  29 (0.7%)  2,319 (58.0%)  791 (19.8%)  6 (0.2%) | 210 (5.5%)  828 (21.8%)  9 (0.2%)  2,013 (53.0%)  737 (19.4%)  1 (0.0%) | 11 (5.7%)  54 (28.0%)  1 (0.5%)  99 (51.3%)  28 (14.5%) |
| Sex   - Female - Male - Unknown | 14,768 (57.8%)  10,792 (42.2%)  1 (0.0%) | 2,241 (56.1%)  1,752 (43.9%)  2 (0.1%) | 2,172 (57.2%)  1,626 (42.8%) 0 (0%) | 98 (50.8%)  94 (48.7%)  1 (0.5%) |
| Race   - Asian - Black / African American - Native American - Native Hawaiian/Pacific Islander - White / Caucasian - Other - Unknown | 623 (2.4%)  659 (2.6%)  92 (0.4%)  19 (0.1%)  23,245 (90.9%)  539 (2.1%)  384 (1.5%) | 58 (1.5%)  79 (2.0%)  12 (0.3%)  4 (0.1%)  3,675 (92.0%)  94 (2.4%)  73 (1.8%) | 87 (2.3%)  78 (2.1%)  14 (0.4%)  3 (0.1%)  3,536 (93.1%)  46 (1.2%)  34 (0.9%) | 4 (2.1%)  3 (1.6%)  1 (0.5%)  0 (0%)  178 (92.2%)  3 (1.6%)  4 (2.1%) |
| Ethnicity   - Hispanic or Latino - Not Hispanic or Latino - Unknown | 1,022 (4.0%)  23,892 (93.5%)  647 (2.5%) | 203 (5.1%)  3,662 (91.7%)  130 (3.3%) | 102 (2.7%)  3,621 (95.3%)  75 (2.0%) | 13 (6.7%)  173 (89.6%)  7 (3.6%) |
| Number of Elixhauser comorbidities   - 0 - 1-4 - 5-9 - 10+ | 17,708 (69.3%)  1,387 (5.4%)  3,805 (14.9%)  2,661 (10.4%) | 2,897 (72.5%)  136 (3.4%)  608 (15.2%)  354 (8.9%) | 2,419 (63.7%)  256 (6.7%)  605 (15.9%)  518 (13.6%) | 126 (65.3%)  11 (5.7%)  34 (17.6%)  22 (11.4%) |
| Dates of second dose   - *Number with 2nd dose* - Earliest - 25th % - Median - 75th % - Latest | 28,082  2021-01-18  2021-02-24  2021-03-23  2021-04-18  2021-12-27 | 3,412  2021-01-19  2021-03-01  2021-03-30  2021-04-23  2021-12-23 | 4,023  2021-01-19  2021-02-22  2021-03-12  2021-04-02  2021-06-17 | 193  2021-01-25  2021-02-18  2021-03-10  2021-04-06  2021-05-20 |
| Time between second dose and test   - Minimum - 25th % - Median - 75th % - Maximum | 0  134  185  231  339 | 1  167  203  239  334 | 135  240  263  287  339 | 161  244  268  298  334 |
| Dates of third dose   - Earliest - 25th % - Median - 75th % - Latest |  |  | 2021-08-13  2021-09-25  2021-10-29  2021-11-10  2021-12-13 | 2021-08-15  2021-09-01  2021-10-28  2021-11-17  2021-12-08 |
| Time between third dose and test   - Minimum - 25th % - Median - 75th % - Maximum |  |  | 14  24  37  54  134 | 14  27  44  63  131 |
